# Recrystallization of tubules from natural lotus (*Nelumbo nucifera*) wax on a Au(111) surface

**DOI:** 10.3762/bjnano.2.30

**Published:** 2011-05-25

**Authors:** Sujit Kumar Dora, Klaus Wandelt

**Affiliations:** 1 Institute of Physical and Theoretical Chemistry, Bonn University, Wegelerstrasse 12, 53115 Bonn, Germany

**Keywords:** AFM, Au(111), lotus wax

## Abstract

We present here the first results on the self-assembly of tubules of natural wax from lotus leaves on a single crystal Au(111) surface. A comparison of the tubule growth on Au(111) to that on HOPG is discussed. Although the tubule formation on both Au(111) and HOPG takes place on an intermediate wax film which should mask the substrate properties, the tubule orientations differ. In contrast to a vertical tubule orientation on HOPG, the tubules lie flat on Au(111). Taking into account the physical properties of HOPG and Au(111), we put forward a hypothesis which can explain the different tubule orientations on both substrates.

## Introduction

Natural nonacosan-10-ol waxes derived from plant leaves have been subjected to numerous studies [1–9]. Electron micrograph studies by Barthlott et al. [7] demonstrated the tubule-like assembly of nonacosan-10-ol molecules on lotus (*Nelumbo nucifera*) leaves, whilst their crystalline nature was verified by X-ray powder diffraction (XRD) and electron diffraction (ED) techniques [10–11]. Koch and co-workers applied tapping mode AFM to study the continuous growth of nonacosan-10-ol tubules on HOPG [8]. By applying a 10 µL droplet of natural wax molecules derived from nasturtium (*Tropaeolum majus*) and lotus (*Nelumbo nucifera*) containing different concentrations of nonacosan-10-ol dissolved in chloroform onto a HOPG surface, they showed for the first time that tubule growth follows a three step mechanism, i.e., a “rodlet → curved structure → tubule”, growth type behavior [8]. By comparing the vertical orientation of tubules on HOPG (non-polar) to horizontally oriented tubules on polar substrates, e.g., silicon, alumina, or glass, they concluded that surface polarity is responsible for tubule orientation. They also demonstrated an increase in the hydrophobicity of the HOPG surface covered with tubules by measuring the contact angle, which increased from 88° (freshly wax covered surface) to 129° after 14 days. A more recent study by the same group [6] demonstrated a number of factors which affect the self-assembly of nonacosan-10-ol molecules on various substrates. By thermal vapor deposition of the wax molecules (in order to exclude solvent influence) they found that at 50 °C (substrate temperature) tubule growth on HOPG occurs rapidly, whereas at 25 °C no tubule was formed on the surface. They also demonstrated that tubules can only be formed from the *S*-enantiomer of nonacosan-10-ol with the addition of a certain amount of diol (0–5%), whereas the *R*-enantiomer produced very few tubular structures even after addition of 4% of the corresponding diol. On the other hand, chemically pure nonacosan-10-ol failed to form any tubules [6]. In addition, a spiral growth mechanism for tubule formation was also proposed in these studies.

Although self-assembly of these tubules was studied on a number of different substrates, as noted above, their growth on single crystal metals has not yet been investigated. Here we report the first study of the growth of tubules from natural wax collected from lotus leaves on a Au(111) surface. In addition, a comparison of tubule growth on Au(111) and HOPG is given in order to obtain a better understanding of the various surface properties affecting the growth kinetics and structure of these tubules. The Au(111) surface was chosen, because gold is regarded as the most chemically inert metal and is a well known substrate for the study of self-assembly of a number of different organic molecules, e.g., long chain alkanes [12–13], both aliphatic and aromatic thiols [14–16], substituted porphyrins [17–18], substituted pyridines [19], 1-nitronapthalene [20], saccharin [21], substituted carboxylic acids [22], polyaniline [23], etc., either by vapor deposition in vacuum, or from solution. The main intention of our study was to demonstrate that tubules from lotus wax can also be reconstituted on single crystal metal surfaces, and compare their kinetics and orientation with materials that have similar surface properties.

## Results

**Figure 1:**
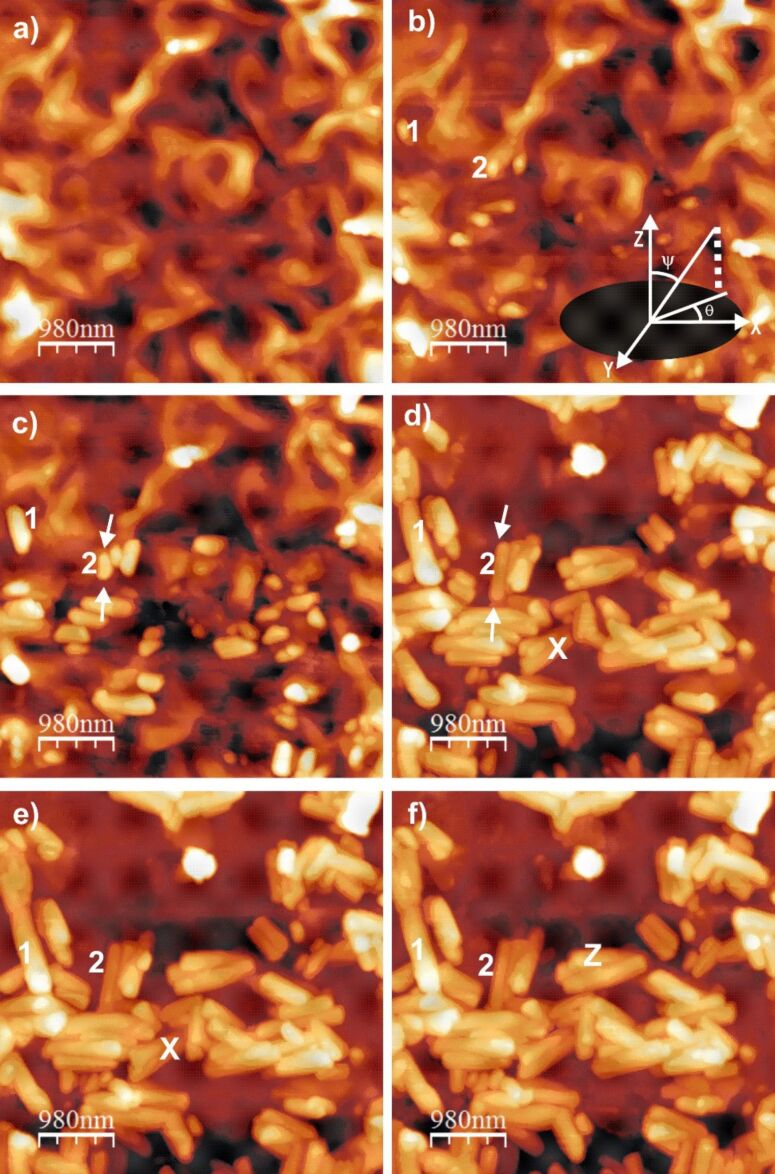
Consecutive AFM images showing nonacosan-10-ol wax tubule growth on a single crystal Au(111) surface about 15–759 minutes after applying 0*.*4 mg*·*mL^−1^ wax solution onto the substrate. Tubule growth starts from a thin film (Figure 1a, 15 minutes) of irregular nature (roughness about 20–300 nm) and rodlets are formed (Figure 1b, 36 minutes) by phase separation. The rodlets then change to tubules (Figure 1c, 77 minutes) before growing longitudinally (see arrows in Figure 1b, Figure 1c and Supporting Information File 1) by the continuous accumulation of wax molecules at both ends (Figure 1d, 207 minutes). Longitudinal growth ceases at *≈*4 hours (Figure 1e, 242 minutes). Further waiting up to 13 hours (Figure 1f, 759 minutes) did not result in any further morphological change. Size = 4*.*9 × 4*.*9 µm^2^, scan rate = 0*.*619 Hz, 256 lines.

Figure 1 shows a series of AFM images of wax growth on the Au(111) surface. A video sequence of all the images taken during scanning, which demonstrates the representative wax crystallization process on Au(111), is available in Supporting Information File 1. As for their polar orientation, the majority of the tubules are oriented in a parallel fashion with respect to the substrate surface: Only a minority do not lie totally flat on the substrate. In other words, these latter tubules have a height difference between their two ends, i.e., they exhibit a slope (for example, the tubule marked 'Z' in Figure 1f). The term polar orientation refers to the tubule orientation with respect to the vertical axis (see inset in Figure 1b, ψ is the polar angle). The average time for completion of tubule formation was about 3–4 hours. The term ”completion” indicates the point where the tubule growth ceases, i.e., when no further changes in dimensions (e.g., width, length) of the tubules are observed. Figure 1a, which was taken 15 minutes after application of the wax solution, shows a thin film of variable roughness (roughness varies between 20–300 nm) covering the entire surface. Figure 1b, which was recorded after 36 minutes, shows the initial phase separation and the formation of rodlets from this thin film. The term phase separation here primarily refers to the isolation of wax molecules which form rodlets (which later on are converted to tubules), from those which do not form rodlets. It is important to note here that these tubules grow on top of a non-tubule forming wax film and not on top of the Au(111) substrate itself. The average time for the onset of this phase separation is about 15–20 minutes. It is worth mentioning that the average time period before the onset of this phase separation is reproducible from a number of experiments carried out under similar conditions which was verified in our case by repeating the experiments three times. The term rodlet used here is actually taken from previous studies by Koch et al [8]. Figure 1c, which was taken after about 77 minutes, shows rodlets undergoing bending, due the continuous incorporation of wax molecules, and hence starting to form tubules. Once the tubules are formed, they start to grow longitudinally at both sides by further addition of wax molecules to either end, as shown by arrows in Figure 1c and Figure 1d (at about 77 and 207 minutes), respectively. The longitudinal growth ceased after about 4 hours (Figure 1e, at about 242 minutes) and no further changes in the dimension of the tubule or the underlying thin film was observed, even although experiments were carried out for up to 13 hours (Figure 1f, 759 minutes). It is also important to note however, that the growth of some of the tubules ceased much earlier, for example, the tubules marked with ”X” in Figure 1d (at about 207 minutes). The reason for such premature saturation might be related to the local unavailability of further tubule forming wax molecules. The average outer diameter of the vast majority of tubules varied between 200–300 nm. Only a negligible number of tubules with an outer diameter of greater than 300 nm were found.

**Figure 2:**
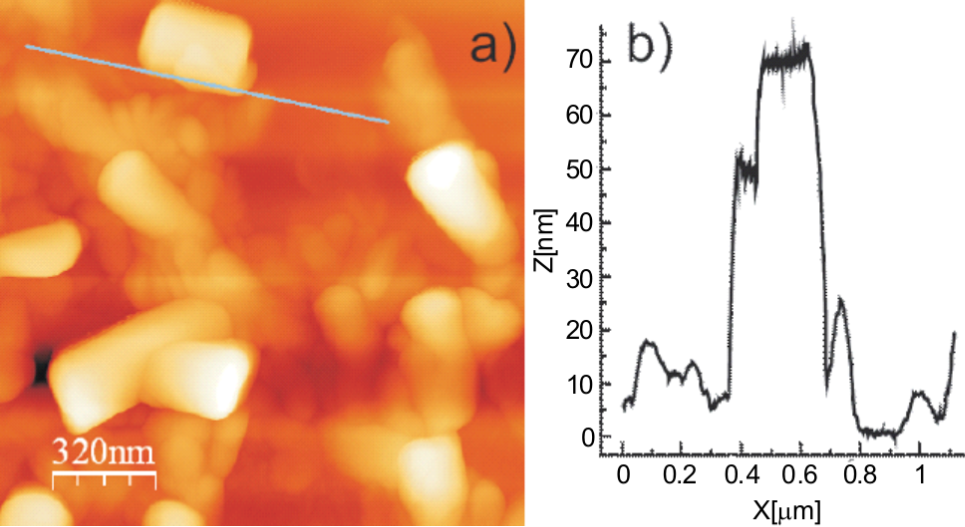
Initial stage formation of lotus wax tubules on a glassy carbon surface (a) and profile across the marked section (b) showing the average layer height to be about 20 nm (standard deviation 2 nm, number of measurements = 5) in agreement with the variation of outer diameters of tubules in multiples of 20 nm. Size = 1*.*6 × 1.6 µm^2^, scan rate = 0*.*519 Hz, 512 lines.

It is interesting to note that the outer diameter of all the tubules varied in multiples of 20 nm (e.g., 200, 220, 260, 280 and 300 nm) as observed in our experiments. Such variations in the outer diameter can also be found on a number of different substrates, e.g., glassy carbon, mica, glass, etc., as was also observed in our experiments. Figure 2a shows an initial stage of layer by layer growth of a tubule on a glassy carbon surface. A line profile across the layer (Figure 2b) clearly demonstrates that the height of a single layer is about 20 nm (standard deviation 2 nm, number of measurements = 5) which clearly agrees with the stepwise tubule outer diameter variation in multiples of 20 nm. The average length of flat lying tubules varies randomly most probably depending upon the local availability of tubule forming wax molecules, but no useful statistics could be drawn from this.

**Figure 3:**
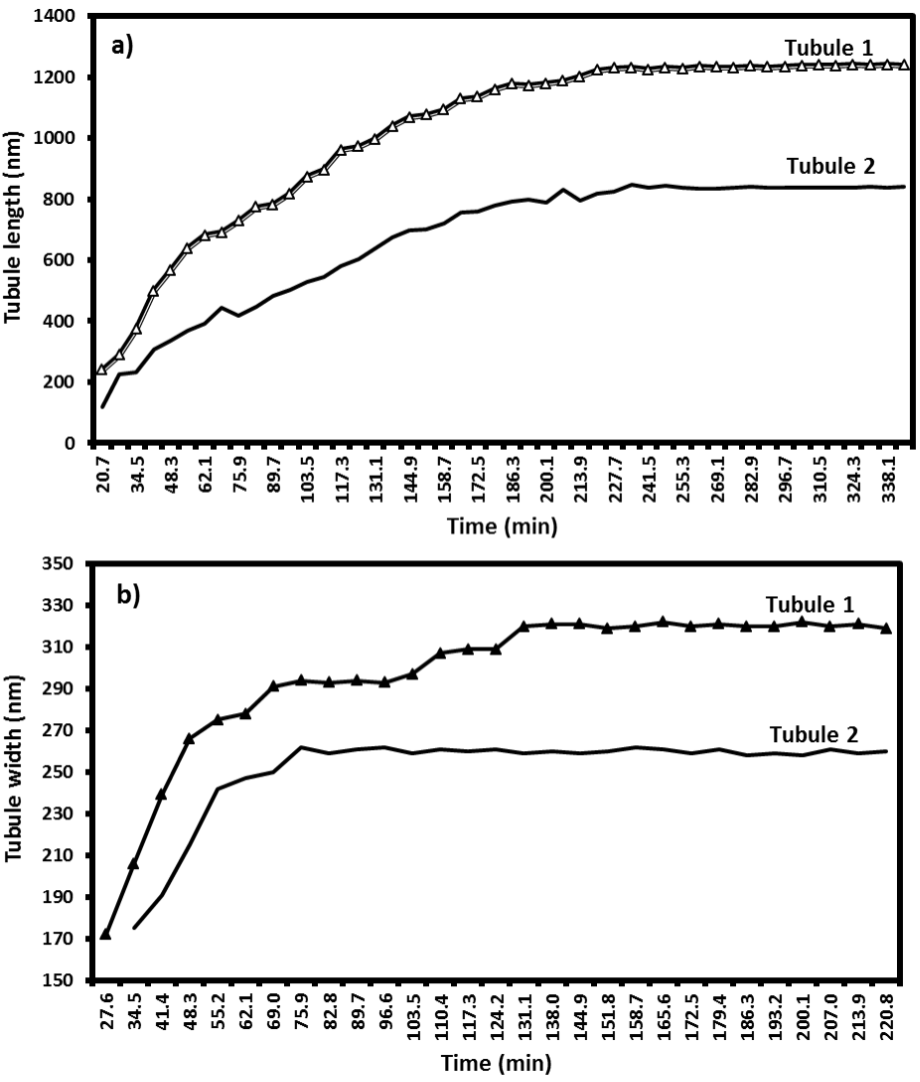
Change in tubule length (Figure 3a) and width (Figure 3b) versus time for two representative tubules (numbered as 1 and 2 in Figure 1) taking into account all the images shown in the video (Supporting Information File 1; partially shown in Figure 1). Tubule growth follows a logarithmic trend and saturates after *≈*4 hours for longitudinal growth, and 2.2 hours or less for radial growth, respectively.

Plots of the increase in length and radial width of some tubules versus time are shown in Figure 3. The curves numbered 1 and 2 refer to the numbers 1 and 2 in Figure 1. The plot (Figure 3a) shows a logarithmic increase in the length reaching saturation after about 3*.*5 hours. Further waiting periods up to 6 hours (and longer - data not shown) did not result in any further measurable change of the tubule length. Comparing the growth of both tubules – 1 and 2 in Figure 1 (plotted in Figure 3a) – it is obvious that the growth rate of tubule 2 was lower and its final length was shorter, but both tubules reached their final length after the same time (about 3*.*5 hours). On the other hand, although radial growth follows a similar logarithmic increase behavior (Figure 3b), the time period of width saturation is considerably lower (about 2.2 hours for tubule 1 and 1.2 hours for tubule 2). This indicates that the tubules continued to grow in length after they reached their final diameter. The term growth indicates increase in both longitudinal as well as radial width of the tubules. This means that tubule 1 was not only longer than tubule 2 but that its radius was also larger (about 320 nm) compared to tubule 2 (about 260 nm). In addition, in no case studied did we find a dependence of the growth kinetics on the concentration of molecules *in solution*. These two observations combined suggest that both the growth rate as well as the saturation length depends on the availability of molecules on the *surface*, or more precisely, within the “capture zone” surrounding each tubule.

## Discussion

**Figure 4:**
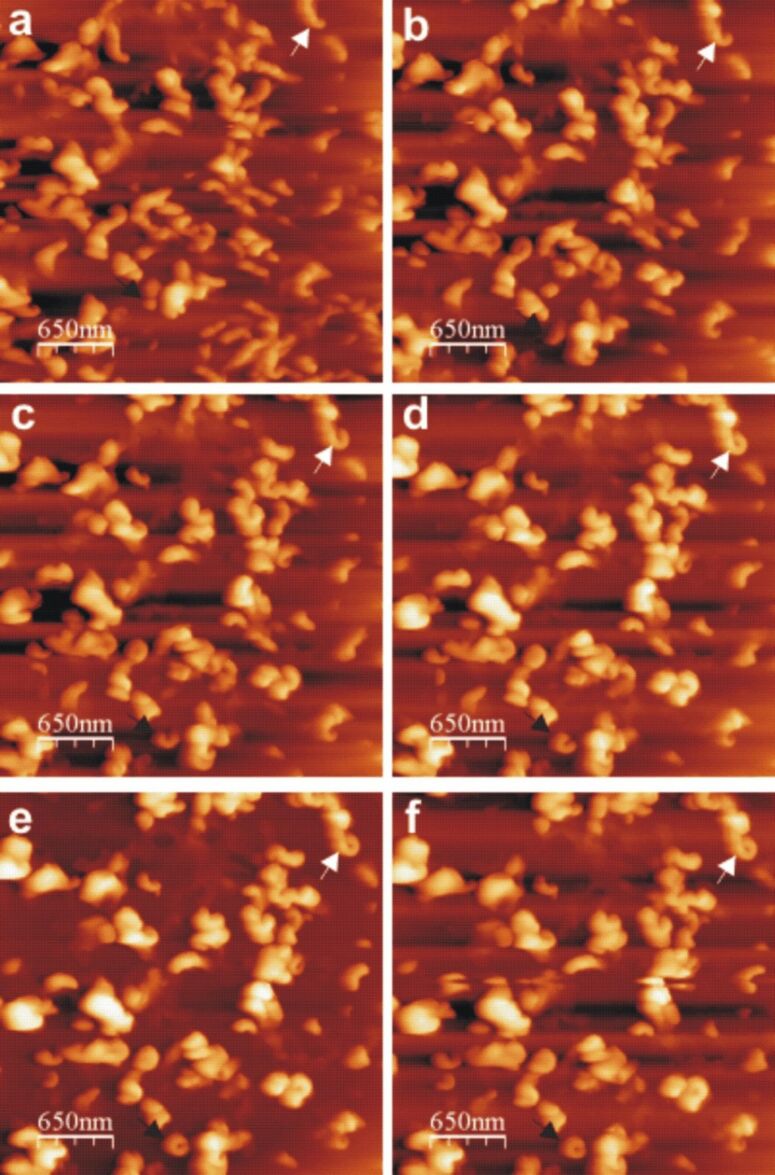
Consecutive AFM images of Lotus (*Nelumbo nucifera*) wax tubule growth on HOPG, about 32–230 minutes after applying 0*.*2 mg*·*mL^−1^ wax solution (chloroform solvent) to the substrate. The arrows show vertical growth of tubules in clockwise (black arrow) as well as anti-clockwise (white arrow) direction on the substrate. The average time for completion of tubule formation is about 3–4 hours. The growth of the tubules starts from rodlets (Figure 3a, 32 minutes) which form curved rodlets (Figure 3b, c, d, e, 66, 98, 148, 180 minutes) before finally forming the complete tubule (Figure 3f, 230 minutes) as marked by arrows. Size = 3*.*25 × 3*.*25 µm^2^, scan rate = 0*.*519 Hz, 512 lines.

As on many other substrates such as glass, mica, and HOPG, we also observed on a Au(111) surface the recrystallization of tubular structures after application of a solution of natural lotus wax in chloroform. AFM images showed that most of the tubules with a polar orientation parallel to the surface reached their final length after approximately 3–4 hours. The reason some of the tubules had a height difference between the two ends was due to their growth over other tubules. Those tubules lying directly on the substrate (or for that matter on the non-tubule forming thin film) did indeed have a parallel orientation with respect to the substrate. This is quite different to the tubules observed by Koch et al on HOPG [9]. Although under similar conditions, the average time period for reaching saturation length is comparable for both HOPG and Au(111), the tubule orientation differs, i.e., a horizontal tubule orientation on Au(111) in comparison to the vertical orientation on HOPG (Figure 4).

Taking into account the physical properties of HOPG and Au(111), where both are non-polar and crystalline in nature, this difference in the orientation of tubules is surprising. It is a well known fact that self-assembly of organic molecules with aliphatic chains results in epitaxial growth on HOPG due to their well matched atomic distances. For example, Watel et al. [24] have shown for long chain alkanes that the carbon skeleton lies parallel to the HOPG surface. On the other hand, epitaxial growth of long chain alkanes on Au(111) resulted in a vertical arrangement of the carbon skeleton [12] on the substrate. Similarly, Dorset et al. [25–26] have also demonstrated the unusual orientation of paraffin waxes by studying their epitaxial growth on organic crystals. It is important to note that epitaxial growth is limited to the first few layers attached to the substrate, however, in our case tubules were formed on top of a thin film (formed from non-tubule forming wax molecules) rather than directly on the substrate surface. The presence of such a thin film on both the Au(111) and the HOPG surface should make both surfaces behave in an equivalent manner and suppress any effect of a direct epitaxial relationship with the respective substrate. Yet, a vertical orientation of tubules could be found on HOPG compared to the horizontal tubule orientation on Au(111). To support our claim, we would also like to emphasize that even after applying a 10 µL droplet of a higher concentrated solution (10 mg·mL^−1^) on to HOPG, vertically oriented tubules were also formed. By contrast, we have also observed rodlets of octacosan-1-ol growing only in a parallel fashion (also reported by Koch et al. [27]) on top of its thin film by applying a 10 µL droplet of 0.4 mg·mL^−1^ octacosan-1-ol in chloroform on to HOPG (Supporting Information File 2). This strongly suggests that the orientation of the three dimensional structure on top of the thin film is independent of the epitaxial behavior. Therefore we can clearly say that none of the above mentioned substrate properties are responsible for the orientation of the tubules on these substrates. This also leads to the question, if none of these properties are responsible then what else is responsible for the vertical tubule orientation on HOPG? The answer might be found in the free standing dangling bonds on HOPG. Many authors have already reported the presence of free standing dangling bonds on the HOPG surface, which is different from metal surfaces [28–30]. The presence of such dangling bonds in HOPG might act as the preferential adsorption site for some particular constituent of the lotus wax thereby influencing the segregation behavior within the wax layer. As a consequence, the resultant surface composition of the wax film may favor vertical growth of the tubules. On the other hand, on Au(111) no such dangling bonds are available and hence the thin film might have a different surface composition upon which tubules grow horizontally. This substrate dependent segregation behavior, and hence, the different resultant concentration profile within the wax film, could be an explanation as to how the influence of the different substrates could be transferred to the surface of the wax film where the tubules actually grow. However, this “chromatographic effect” of the substrate is purely a hypothesis and no data is available yet to support this hypothesis. As for the azimuthal orientation of tubules, we would also like to make it clear that the tubules are oriented in a random manner and do not follow the symmetry found on a gold surface. The term azimuthal orientation refers to the tubule orientation with respect to the azimuthal angle in the X–Y plane (see inset in Figure 1b, θ is the azimuthal angle). Such random orientation of tubules can also be found on non-crystalline substrates, e.g., glass, glassy carbon, etc.

The next interesting behavior of the tubules is the variation of their outer diameter in multiples of 20 nm. To make sure image artefacts did not affect our measurements, we applied a very slow scanning rate of 0.619 Hz. In addition, tubule diameters were measured with respect to the flat surface and the height measurement in Figure 2 clearly demonstrates the single layer height to be about 20 nm. All these factors strongly support our measurements of outer tubule diameter as being in multiples of 20 nm. Taking into account the molecular length of nonacosan-10-ol, which is about 4 nm long (assuming all methylene groups are in trans-configuration [31]), it can be assumed that a single layer of such a tubule consists of five layers of nonacosan-10-ol (or for that matter corresponding -diol) molecules. The reason for the stability of an individual tubule layer consisting of five molecular layers of nonacosan-10-ol is still unknown, but a high resolution microscopic study might shed more light on the exact molecular architecture within the layers.

The bending of thin films to tubules most probably happens in order to release stress that arises due to the increase in length and thickness of the individual rodlets. Numerous studies have described structural transitions in thin films which contribute to the relaxation of stress at their interface [32–34].

Another interesting observation is the rather uniform and confined diameter of the completed tubules of *<*300 nm. This, in turn, must be an inherent property of the molecular architecture of the wax tubules. The incipient radial growth of the tubules occurs stepwise in layers of 20 nm thickness. Their molecular arrangement seems to favor a certain curvature of such a 20 nm thick layer. However, with the increasing diameter of a tubule, this curvature decreases leading to the accumulation of internal stress. As a consequence, at a tubular diameter of about 300 nm the curvature of the next added layer of 20 nm would be too low, and hence the internal stress too high, so that no further layer will grow around the tube.

To conclude we would also like to refer to the orientation of wax tubules found in previous studies of the self-assembly of natural nonacosan-10-ol wax on different substrates. Jetter and co-workers [2] showed that substrate polarity and roughness play no role in tubule crystallization by demonstrating a parallel orientation of tubules on a number of different substrates, e.g., polyethylene, polypropylene, Teflon, alumina, mica, glass, etc. However, they applied a larger volume of solution of 50–250 µL (10 mg*·*mL*^−^*^1^) which effectively masks any effect of the substrate. A more recent study by Koch et al. [9] demonstrated the effect of substrate polarity on the tubule growth by showing a vertical orientation on HOPG and a horizontal orientation on silicon and alumina. However, in our study by combining various properties (e.g., polarity, crystallinity) together at two different surfaces we have demonstrated that none of these properties is directly responsible for tubule orientation. To support our data, we would also like to emphasize that the self-assembly of long chain alcohols, e.g., octacosan-1-ol, on HOPG as well as on other materials, e.g., glass, mica, or silicon, results in a totally different arrangement on HOPG compared to all other materials, as demonstrated by Dommisse [1] and Hommes [35]. This reinforces the notion that some very special properties of HOPG are responsible for the entirely different self-assembly process on its surface and a hypothetical explanation is given above. This raises also the question whether HOPG is in fact a useful substrate in order to mimic natural leaf surfaces, which is actually often done, because on natural leaves no such preferentially vertical orientation is found [7].

## Conclusion

We have performed the first study of the growth of nonacosan-10-ol tubules on a single crystal Au(111) surface. By comparing the wax growth on Au(111) with that on a HOPG substrate, being both non-polar and crystalline, we have found that none of these properties are responsible for tubule orientation. From this study, we have also concluded that HOPG most probably has some special properties that lead to the unusual upright orientation of tubules on its surface. We have proposed as a hypothesis that ultimately the presence of free-standing dangling bonds on HOPG, and thereby a substrate specific segregation behavior within the wax materials, might be responsible for this tubule orientation. Furthermore, we propose the buildup of internal stress within the layered structure of the tubules which limits the outer diameter of tubules to approximately 300 nm. Finally, we have raised the question of whether or not HOPG is a suitable material to mimic wax growth as on natural leaf surfaces.

## Experimental

The nonacosan-10-ol wax materials, which were extracted with chloroform from lotus (*Nelumbo nucifera*) leaves, were obtained from the Nees Institute for Biodiversity of Plants at Bonn University. These wax materials, which were used in all our experiments, are actually a mixture of long chain hydrocarbons and their derivatives, e.g., alcohols with one or two OH-groups, aliphatic acids, as well as some unidentified components. For the exact chemical composition of this wax, the reader is referred to the following article [8]. A concentration of 0*.*4 mg*·*mL*^−^*^1^ of wax molecules was prepared by dissolving 2 mg of wax in 5 mL of chloroform. The Au(111) single crystal used in our experiments was bought from Mateck GmbH, Jülich, Germany. The Au(111) crystal was cleaned by flame annealing (a well established procedure in electrochemical surface science). For this purpose, the crystal was placed on a ceramic plate and annealed with a butane gas flame for 3 minutes up to faint red glow (600–700 °C). The crystal was then cooled to room temperature in an argon atmosphere for about 15 minutes. A clean HOPG surface was prepared by simply removing a few atomic layers with adhesive tape.

AFM measurements were carried out with a Picoscan AFM (Molecular Imaging, Tempe, AZ, USA) with a PicoScan controller coupled with a MAC Mode controller. The system was operated in MAC Mode AFM employing type I MAC levers under ambient conditions. The type I MAC levers are silicon cantilevers with a length of 90 µm and a typical tip radius of less than 10 nm, having a resonance frequency of 75 kHz and a force constant of 3 N/m (NanoAndMore GmbH, Wetzlar, Germany). Appropriate imaging conditions were a scan size of 4.9 × 4.9 µm^2^, a scan rate of 0.5–1 lines/s, and an image size of 256 × 256 pixels. In order to minimize interaction between tip and sample, the set point was chosen close to the upper limit. All experiments were performed at room temperature (20–24 °C).

For real time observations of wax recrystallization by AFM, a 10 µL droplet of wax molecules dissolved in chloroform was applied onto the central part of both the Au(111) and a HOPG surface. The total area of wax crystallization was about 20 mm^2^ for both substrates. The chloroform took ca. 30 seconds to evaporate from the surface leaving the wax molecules on the substrate. The substrates were then fixed to the AFM stainless steel base plate, and the first image acquisition started as soon as possible after the chloroform evaporation, typically after 8–10 minutes. AFM images were taken consecutively from the same substrate area over a period of several hours applying a constant scan rate (as denoted in the respective figure captions). Height and length measurements of the resultant wax crystals were made with the program WSxM (Version 3.0; Nanotec Electronica, Madrid, Spain).

## Supporting Information

File 1 Natural nonacosan-10-ol wax crystallization on Au(111) surface.

File 2 Parallel orientation of 3-dimensional structures of octacosan-1-ol on HOPG by recrystallization from chloroform solution.
