# Supplementary figures and images for: Recrystallization of tubules from natural lotus (Nelumbo nucifera) wax on a Au(111) surface
Source: Beilstein J Nanotechnol. 2011 May 25;2:261–7. doi: 10.3762/bjnano.2.30 (PMC3148047; doi:10.3762/bjnano.2.30)

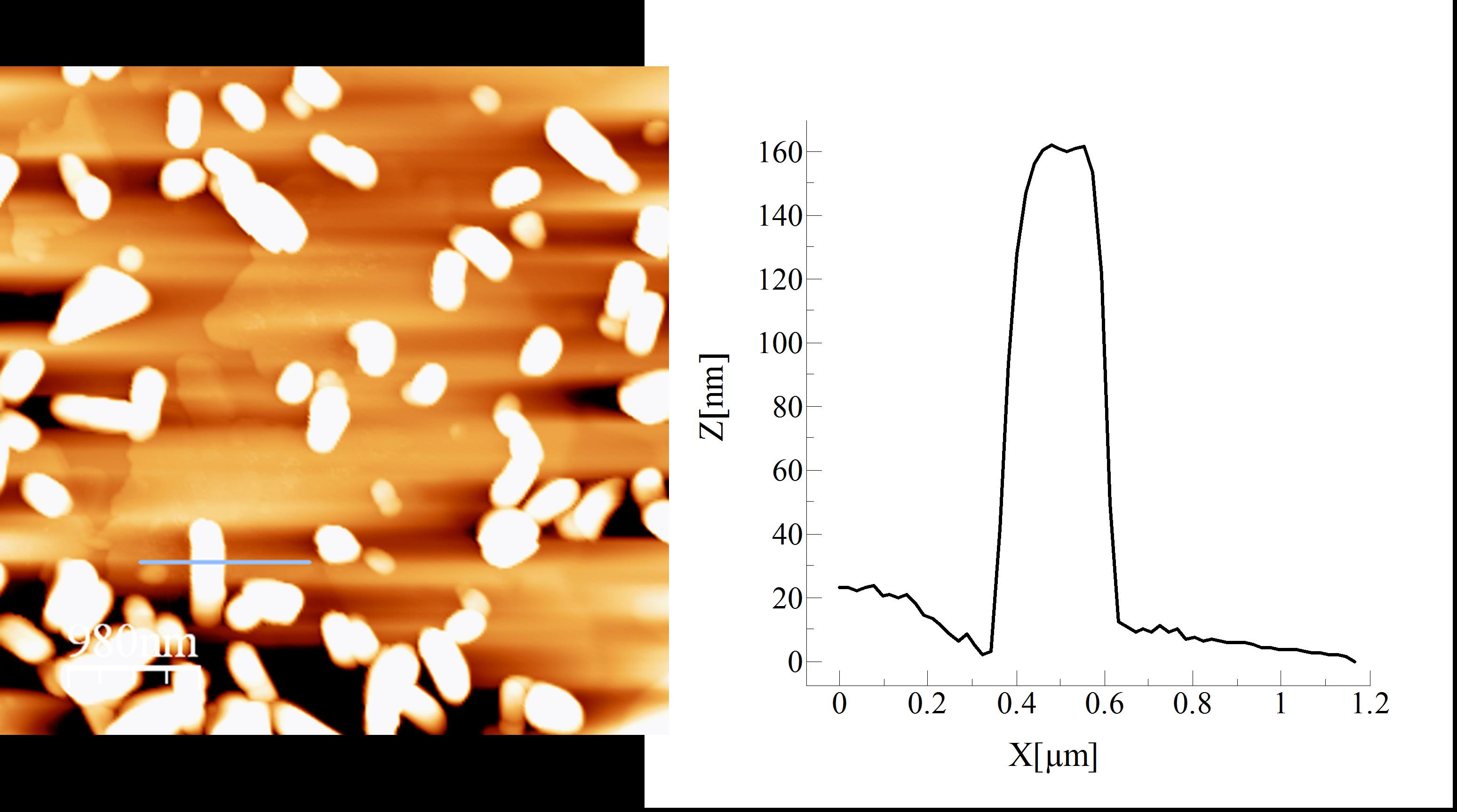

Supplement: File 2 — Parallel orientation of 3-dimensional structures of octacosan-1-ol on HOPG by recrystallization from chloroform solution. [file Beilstein_J_Nanotechnol-02-261-s002.jpg]
